# Granulocytes: New Members of the Antigen-Presenting Cell Family

**DOI:** 10.3389/fimmu.2017.01781

**Published:** 2017-12-11

**Authors:** Ang Lin, Karin Loré

**Affiliations:** ^1^Department of Medicine Solna, Immunology and Allergy Unit, Karolinska Institutet (KI), Solna, Sweden; ^2^Center for Molecular Medicine, Karolinska Institutet (KI), Stockholm, Sweden

**Keywords:** antigen presentation, MHC, neutrophil, eosinophil, basophil

## Abstract

Granulocytes, the most abundant types of leukocytes, are the first line of defense against pathogen invasion. However, the plasticity and diversity of granulocytes have been increasingly revealed, especially with regard to their versatile functions in orchestrating adaptive immune responses. A substantial body of recent evidence demonstrates that granulocytes can acquire the function as antigen-presenting cells under pathological or inflammatory conditions. In addition, they can acquire surface expression of MHC class II and costimulatory molecules as well as T cell stimulatory behavior when cultured with selected cytokines. The classic view of granulocytes as terminally differentiated, short-lived phagocytes is therefore changing to phenotypically and functionally heterogeneous cells that are engaged in cross-talk with other leukocyte populations and provide an additional link between innate and adaptive immunity. In this brief review, we summarize the current knowledge on the antigen-presenting capacity of granulocyte subsets (neutrophils, eosinophils, and basophils). Underlying mechanisms, relevant physiological significance and potential controversies are also discussed.

## Introduction

As key components in the innate immune system, granulocytes have generally been considered as rapid responders in the first line of defense against pathogens. The role of granulocytes has long been considered restricted to the initial phase of the defense. However, a substantial body of evidence has indicated that there is a functional heterogeneity and plasticity among granulocytes, with most emphasis on their versatile abilities in shaping adaptive immune responses (1, 2). To this end, T cell responses orchestrated by granulocytes *via* antigen presentation have been described and received considerable attention. The increasingly recognized antigen-presenting function of granulocytes has led to the suggestion that they should be referred to as atypical antigen-presenting cells (APCs) (3). In this review, we will focus on the three main granulocyte subsets (neutrophils, eosinophils, and basophils) and summarize current knowledge about their role as APCs in experimental and clinical systems. We will also discuss the potential underlying mechanisms and their physiological significance.

## Classical Features of APCs

The professional APC family consists of dendritic cells (DCs), B cells, and monocytes/macrophages, among which DCs are the most potent due to their superior ability to prime naïve T cells. To be classified as a professional APC, a cell should have the ability of antigen acquisition and processing as well as exhibit accessory molecules allowing them to interact with T cells. The so-called “three-signal model” (4) is usually used to define the APC function required for the activation of T cells. Engagement between the MHC–peptide complex and T cell receptor provides *signal 1*. While this is necessary it is not sufficient; and *signal 2* is thus required, which is delivered through interactions between costimulatory receptors and complementary ligands on T cells. In addition, activated APCs can secrete various cytokines as *signal 3* that drive the polarization of T cells into different effector cells. Typical APCs need to have at least *signal 1* and *2* to have the capacity to stimulate T cells. The cytokine-priming function by *signal 3* primarily determines the nature of the T cell responses generated.

## Granulocytes as APCs: Licensed or Not?

The initial notion of an antigen-presenting function existing in granulocytes stems from the cells having the ability to internalize antigens and possessing the basic molecular machinery required for antigen presentation (5). Despite this, discrepancies exist with respect to how the properties of granulocytes change under different circumstances and the models used. To this end, resting neutrophils show no or very low expression of MHC-II and have been shown to be unable to stimulate proliferation of naïve CD4^+^ T cells in a mixed lymphocyte reaction (6). This suggests that neutrophils are unable to, or at least inefficient at priming naïve T cell responses, which is in contrast to classical APCs. It was reported that as few as 210–340 MHC-II molecules were sufficient for a cell to act as an APC (7). Whether granulocytes can act as APCs is therefore likely determined by the microenvironment that the cells are exposed to. It is also important to note that how this function influences adaptive immune responses *in vivo*, especially in humans, needs investigation.

## Neutrophils as APCs

Neutrophils are the dominant population among granulocytes. The contribution of neutrophils in adaptive immunity has been to a large extent undervalued due to the long-standing paradigm that they are terminally differentiated short-lived cells. However, this dogma is starting to change with the emerging evidence that neutrophils can survive much longer than previously thought, especially under inflammatory conditions or during interactions with other cell subsets (8, 9). The circulatory half-life of neutrophils under homeostasis appears to be between 13 and 19 h (10). Moreover, neutrophils inherently express or can *de novo* produce the receptors needed for antigen presentation (11–13). These observations support that neutrophils have the capacity to function as APCs.

### APC Features Induced in Neutrophils by Cytokine Exposure

The original hypothesis that neutrophils can acquire an antigen-presenting function is based on the observations that MHC-II and costimulatory molecules (e.g., CD80 or CD86) can be induced on their cell surface by exposure to specific cytokines such as IFN-γ or GM-CSF (13). Furthermore, it was demonstrated that cytokine-exposed neutrophils gained the ability to stimulate T cells in an MHC-II-restricted manner (14). Human and murine bone marrow neutrophils exposed to GM-CSF can differentiate into neutrophil–DC hybrids, exhibiting a DC-like phenotype and antigen-presenting function, while still maintaining several neutrophil features (15). Both immature and mature neutrophils in mice can acquire DC-like properties, which is in line with both human mature neutrophils and immature neutrophil precursors acquiring DC-like characteristics after exposure to cytokines (GM-CSF, IFN-γ, IL-4, and TNF) (16, 17). Therefore, the plasticity of neutrophils to become APCs may not be restricted to a particular stage of differentiation. In addition to the findings that neutrophils can differentiate to APCs in cytokines-conditioned cultures, neutrophils isolated from patients receiving GM-CSF or IFN-γ treatment have shown well-detectable MHC-II expression (18–20). Induction of MHC-II on neutrophils was also observed in patients with chronic inflammatory diseases associated with high levels of cytokines; for example, in patients with rheumatoid arthritis or Wegener’s granulomatosis (11, 21, 22).

### Antigen-Presenting Capacity Mediated by the Presence of T Cells

**Figure 1:**
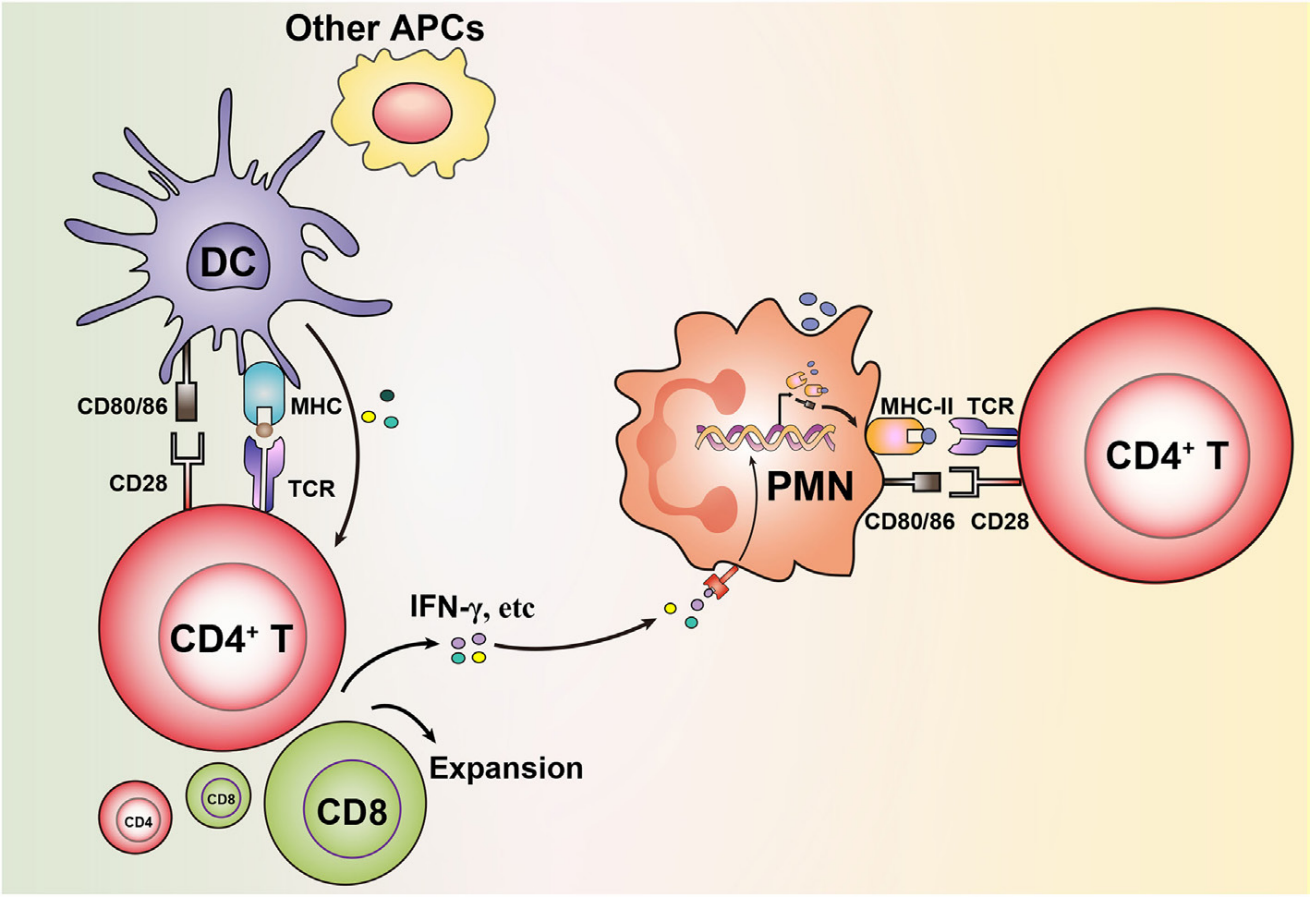
Granulocytes acquire antigen-presenting cell (APC) features *via* bystander activation from neighboring professional APCs stimulating T cells. Antigen presentation initiated by dendritic cells (DCs) or other professional APCs leads to stimulation of T cells to produce cytokines (e.g., IFN-γ). This results in that granulocytes upregulate expression of MHC-II and costimulatory molecules on their surface, which enables them in turn to present antigens to T cells.

Recent studies demonstrated that T cells can deliver signals to neutrophils to differentiate into APCs (23). Fresh human neutrophils cocultured with T cells were shown to acquire surface expression of CD80 and CD86 (14). Neutrophils without any stimulation can induce proliferation of antigen-specific T cells when cultured together with T cells and antigens (14). Murine neutrophils purified from peritoneal exudate cells were shown to express MHC-II and costimulatory molecules after coculture with T cells. Neutrophils isolated from this site could also present OVA peptide antigens to OVA-specific T cells *in vitro* without exogenous cytokines (24). This raises the question of whether the induction of an APC function in neutrophils is in fact initiated by interaction with activated T cells. The very first trigger of neutrophil differentiation may be dependent on bystander activation from adjacent APCs, that present antigens and result in stimulation of antigen-specific T cells, that in turn produce cytokines which subsequently differentiate neutrophils. Upregulation of MHC-II and costimulatory receptors at sufficient levels to present antigen may therefore be induced on neutrophils only if they are present in a milieu where activated T cells secrete cytokines as a consequence of responding to antigen presentation by neighboring APCs (Figure 1). This is supported by that neutrophils exposed to supernatants from cytokine-producing T cells upregulate MHC-II and costimulatory receptors (6). It is well known that toll-like receptor (TLR) activation of DCs promotes their antigen-presenting ability (25). Neutrophils express the majority of TLRs and can be activated *via* TLR ligation (26). However, we recently showed that TLR activation of neutrophils was not sufficient to induce HLA-DR despite upregulation of other markers such as CD11b and CD83 (6).

### Regulation of the Induction of the Antigen-Presenting Function

Although the classical receptors involved in antigen presentation are absent on the surface of resting neutrophils, studies have showed that normal human neutrophils contain cytoplasmic CD80 and CD86 stored in secretory vesicles or granules (11). Cytoplasmic MHC-II in neutrophils has also been detected, although only in about 10% of healthy donors (27). MHC-II, CD80, and CD86 can rapidly translocate to the cell surface upon stimulation of neutrophils (11, 28). In addition to intracellular storage, *de novo* synthesis of MHC-II can also be induced by cytokine exposure, i.e., IFN-γ as described earlier. This IFN-γ-induced MHC-II expression is mediated by the induction of the independent promoter IV, which regulates expression of the class II transactivator, a master regulator controlling MHC-II transcription, as recently reviewed in Ref. (29).

As discussed earlier, neutrophils have been shown to act as APCs after coculture with antigens and memory T cells. Blocking MHC-II on neutrophils was shown to abolish their antigen-presenting function (6, 14). However, as discussed earlier, it is not entirely clear how memory T cells activate neutrophils to express MHC-II. We recently showed that fresh human neutrophils can present cognate protein antigens (cytomegalovirus pp65 or influenza hemagglutinin) to autologous antigen-specific CD4^+^ T cells (6). Acquisition of MHC-II and costimulatory molecules on neutrophils in this system required both the presence of the antigen-specific memory T cells and the specific antigens. While it is plausible that antigens alone could induce neutrophil activation to gain APC function, this appears to be less likely since neutrophils cultured with antigens or even strong stimuli such as TLR agonists do not show significant upregulation of MHC-II (6). The presence of T cells may therefore be required to deliver signals to neutrophils, either by cell-to-cell contact or secretion of mediators, to induce proper cell differentiation to become APCs. Among the T cell-derived cytokines, IFN-γ, in particular, has been shown to be potent at inducing neutrophil differentiation as mentioned earlier (2, 23). Importantly, the antigen-presenting function of neutrophils appears to be provided exclusively by antigen-specific memory T cells and not by naïve T cells. Memory T cells produce IFN-γ more rapidly and in larger quantities. They also have less stringent requirements for activation than naïve T cells such as needing lower levels of costimulatory signals (30). Beyond this, memory T cells may not be fully quiescent cells, especially in situations with inflammation or persistent stimuli present in the microenvironment. In addition, detectable mRNA levels of IFN-γ have been reported in human resting neutrophils (27). A constitutive storage of IFN-γ proteins has also been observed in resting neutrophils, which is spontaneously released during culture or upon stimulation (31). Neutrophils may therefore activate themselves by IFN-γ produced in an autocrine manner.

**Figure 2:**
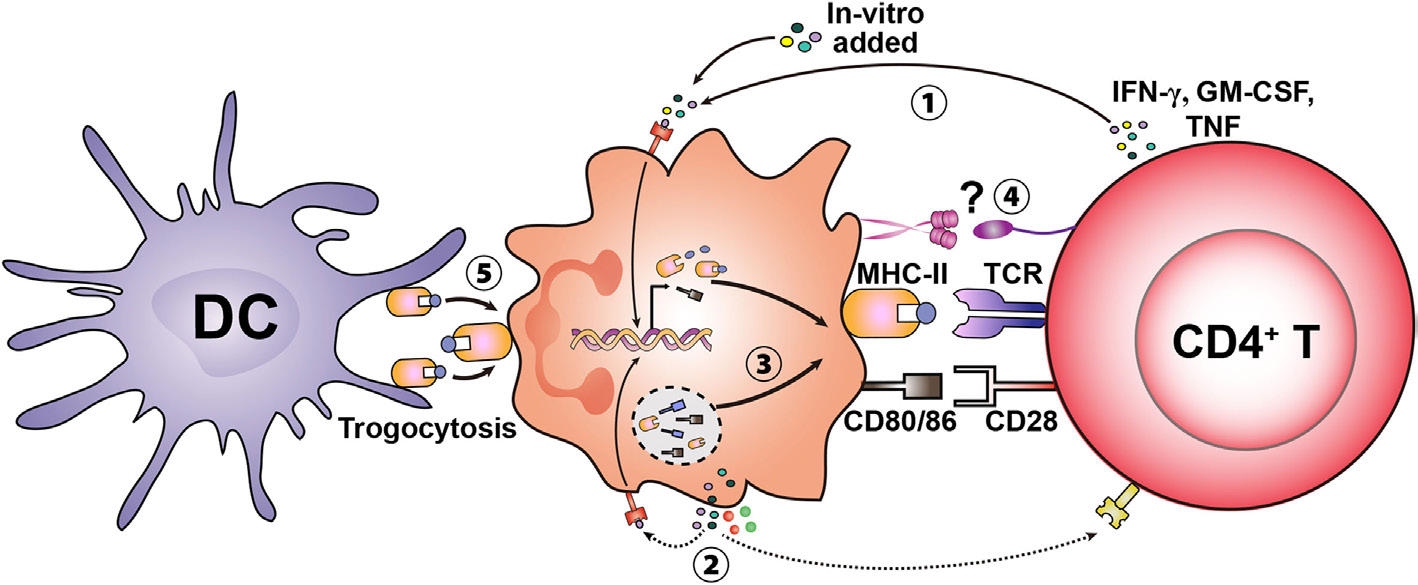
Potential mechanisms involved in inducing the antigen presentation capacity in granulocytes. Granulocytes can *de novo* synthesize MHC-II and costimulatory molecules upon stimulation by exogenous cytokines (e.g., IFN-γ, GM-CSF, and TNF) likely derived from activated T cells ①. Cytokines produced in an autocrine manner may also activate granulocytes and induce the antigen-presenting cell (APC)-associated machinery ②. In addition to *de novo* synthesis, neutrophils contain intracellular stores of MHC-II and costimulatory molecules, which translocate to the cell surface upon stimulation ③. Specific receptors expressed on granulocytes and T cells may mediate initial cell-to-cell contact and amplify the differentiation of granulocytes to become APCs ④. Basophils have also been shown to acquire MHC–peptide complexes from other APCs [e.g., dendritic cells (DCs)] through trogocytosis (transfer of MHC-II–peptide complexes) leading to presentation to T cells ⑤.

In addition to secreted mediators from memory T cells, a direct cell-to-cell contact between T cells and neutrophils may be sufficient to induce the antigen-presenting function of neutrophils. In a recent study, T cell-induced expression of MHC-II on neutrophils was abolished when neutrophils and T cells were separated by a transwell system (24). The receptors that are critical in the T cell–neutrophil interaction remain as yet unknown. However, the classical costimulatory receptor–ligand interactions that facilitate the formation of the immunological synapse are potential candidates. As discussed with IFN-γ production, memory T cells express comparatively higher levels of several receptors than naïve T cells, which may again explain the inability of the naïve T cells to induce phenotypic differentiation of neutrophils. For example, memory T cells express higher levels of intercellular adhesion molecule I (ICAM-1) than naïve T cells (32). ICAM-1 predominantly binds to the integrin molecule Mac-1 (CD11b/CD18), which is constitutively expressed by neutrophils. CD66b, a molecule expressed exclusively on neutrophils can function as a receptor for galectin-3, which is constitutively expressed by human CD4^+^ memory T cells but only at a low level on naïve T cells (33–35). The aforementioned receptor–ligand interactions between memory T cells and neutrophils may provide the requisite signals to initiate neutrophils to express MHC-II on their surface. With expression of MHC-II on neutrophils, there will be a further amplification of the MHC–TCR ligation that would activate more T cells to secrete cytokines, and augment MHC-II expression on neutrophils. This positive feedback loop may be central for induction and maintenance of antigen presentation by neutrophils. This potential mechanism may also apply to other granulocyte subsets (Figure 2).

Details on the pathways involved in antigen processing in neutrophils remains largely unknown. MHC-II associated invariant chain (Ii), one of the critical components binding to nascent MHC-II molecules to regulate the loading of MHC-II with peptides during antigen processing, has not been detected in neutrophils (36). However, cathepsin S, the lysosomal enzyme that degrades Ii to facilitate MHC-II-mediated antigen presentation, was shown to be expressed in neutrophils (37). In addition, the formation and maturation of phagosomes in neutrophils differ from classical APCs (i.e., macrophages) in many respects (38). Therefore, whether the phagocytic and endocytic pathways that are involved in MHC-II-mediated antigen presentation by classical APCs are fully operational in neutrophils remains to be investigated. Aside from the molecular mechanisms, the types of antigens that neutrophils are able to process and present also need further clarification. As described earlier, neutrophils can present soluble protein or peptide antigens to CD4^+^ T cells. Limited evidence is available regarding presentation of particulate antigens or immune complexes such as whole microorganisms, virosomes, or antigens formulated in nanoparticles *via* MHC-II, although this seems quite likely to occur. It has been shown that neutrophils can cross-present particulate bead-conjugated OVA antigens as well as heat-killed bacteria to induce CD8^+^ T cell responses (39, 40).

### Physiological and Pathological Significance

Although the antigen-presenting function of neutrophils has been well documented in experimental settings, it is not clear to what extent their relative contribution is compared to other classical APCs. Consequently, there is still much left to understand regarding the physiological or pathological significance. It is clear that there are conditions and treatments associated with inflammation and high levels of cytokines that result in neutrophils expressing an APC feature; but whether this also holds true under normal circumstances needs more investigation. Clinical findings showed an upregulation of MHC-II on neutrophils in patients with active Wegener’s granulomatosis or rheumatoid arthritis (21, 22). Since aberrant expression of MHC-II is associated with development of autoimmune diseases (41), a possibility is that neutrophils play a role in the pathogenesis of the disease by presenting autoantigens.

Infiltration of neutrophils to inflammatory sites and their rapid migration to secondary lymphoid organs [i.e., draining lymph nodes (dLNs)] have been reported in different contexts (42). In BCG-immunized mice, neutrophils were the predominant cells to capture BCG at injection sites and transport the vaccine to T cell areas in dLNs (43). Migration of antigen-bearing neutrophils from peripheral sites to dLNs likely contributes to T cell stimulation. We recently evaluated this after vaccination in non-human primates and found that neutrophils represented the largest population of cells internalizing vaccine antigens at both the vaccine injection site and in the vaccine dLNs (6, 44). Vaccine^+^ neutrophils in the dLNs could present the vaccine antigen to memory CD4^+^ T cells. Although DCs and monocytes demonstrated a superior antigen-presenting capacity, the overall impact of neutrophil-derived responses may be substantial since the neutrophils vastly outnumber the conventional APCs. An indication of neutrophils being able to differentiate into APCs in a transient inflammatory state is that neutrophils isolated from vaccine-draining dLNs showed higher MHC-II expression than neutrophils isolated from contralateral control dLNs from the same animal (6, 44).

In addition to conventional antigen presentation *via* MHC-II, there is also evidence of the ability of cross-presentation by neutrophils (40). Studies have demonstrated that the machinery and pathways associated with antigen cross-presentation are operative in neutrophils (45–47). Human circulating neutrophils isolated from patients with acute sepsis could cross-present proteins to CD8^+^ T cells (48). In addition, tumor-infiltrating neutrophils isolated from patients with lung cancer could cross-present tumor antigens to promote anti-tumor T cell responses (49). Based on these data, a cross-presenting function in neutrophils may also be induced under special circumstances and elevate the importance of these cells in maintaining robust T cell responses.

## Eosinophils as APCs

Eosinophils are well known as effector cells combating parasitic infections and as important mediators of allergic responses. Recently, the role of eosinophils as APCs has also been proposed. Eosinophils can internalize and process antigens as well as shuttle them to lymphoid tissues (50, 51). MHC-II molecules are absent on resting eosinophils but can be induced by cytokines (52, 53). Furthermore, cytokine-exposed eosinophils can present protein antigens as well as superantigens to T cells in an MHC-dependent fashion (53, 54). In contrast to neutrophils, eosinophils pulsed with parasite antigens were shown to not only present to memory T cells but also prime naïve T cells and induce their Th2 polarization (55). Further evidence was provided by the same group using an *in vivo* model, with antigen-pulsed eosinophils injected into naïve mice. It showed that eosinophils could prime naïve mice for Th2 responses generating a robust T cell proliferation and antigen-specific antibody titer (56). However, there are conflicting reports showing that eosinophils are unable to prime naïve T cells (57), although this has been challenged (53) because ammonium chloride (NH_4_Cl) was used for red blood cell lysis, which can suppress antigen processing by inhibiting lysosomal enzyme activity (58, 59). Overall, the role of eosinophils as APCs has been well supported and recognized. A series of studies reported that eosinophils express MHC-II and costimulatory molecules in different compartments such as blood and sputum of asthmatics as well as bronchoalveolar lavages from patients with allergic airway diseases (60–62). Eosinophils recruited to the airways may play an important role in presenting particulate aeroallergens due to their superior ability over DCs and B cells to phagocytize particulate antigens (51). In addition, the expression of a range of immunoglobulin receptors on eosinophils may facilitate uptake of antigens present as immune complex (63). The antigen-presenting capacity of these cells, however, remains to be further elucidated. One may speculate that the APC capacity of eosinophils may play a role in the initiation or amplification of allergic immune responses and possibly contribute to disease severity.

## Basophils as APCs

Basophils represent a very rare population (less than 1%) of circulating leukocytes. Their central role in allergic and parasitic conditions has been widely recognized. Studies recently demonstrated that basophils are superior than DCs at inducing polarized Th2 responses (64). In mice challenged with protease allergens or helminthic parasites (65, 66), basophils were shown to acquire the APC machinery, rapidly migrate to dLNs and present antigens as well as produce IL-4 to drive Th2 polarization. Surprisingly, depletion of CD11c^+^ DCs did not affect Th2 differentiation and MHC-II-deficient mice adoptively transferred with antigen-loaded MHC-II^+^ basophils still showed robust Th2 responses. This implies that basophils alone could provide sufficient signals to prime Th2 responses, while DCs were insufficient and dispensable. In addition, murine basophils were reported to present peptide antigens or even cross-present protein antigens to naïve CD8^+^ T cells and drive their differentiation into IL-10-producing effector cells (67). It was shown that basophils were insufficient at taking up particulate antigens, suggesting that their antigen-presenting function may be restricted to soluble antigens (66). To date, studies in mice have provided several pieces of convincing data supporting that basophils can act as APCs. However, in the human immune system, contradictory findings have been reported. Fresh human basophils do not show detectable mRNA or protein levels of either MHC-II or costimulatory molecules, but these receptors can be induced by cytokines as discussed above for neutrophils and eosinophils. However, the cytokine-driven expression of APC machinery on human basophils did not result in functional antigen presentation (68). It was shown that cytokine-exposed basophils isolated from healthy subjects lack the ability to present antigens (69). Moreover, studies using basophils from allergic patients failed to detect their antigen-presenting function (70). This may be due to insufficient antigen loading onto MHC-II caused by limited expression of MHC-II associated invariant chain (Ii) and cysteine endoprotease cathepsin S in human basophils (71). In addition, cytokine-exposed basophils were also shown to be incapable of presenting peptide antigens (68). Therefore, there is no clear evidence yet in support of human basophils being able to act as APCs in contrast to their murine homologs. However, it is still early to draw definitive conclusions. A very recent study showed that murine basophils acquired antigen-presenting function by trogocytosis, which is a dynamic process where intercellular cell surface proteins are transferred between cells; in this case, a basophil acquiring MHC–peptide complexes from an APC facilitated by ICAM-1–LFA-1 ligation (72). This provides a novel mechanism that may also be deployed by human basophils. Moreover, upregulation of MHC-II on basophils from different compartments (blood, LN, and spleen) was reported on patients with systemic lupus erythematosus (73). Therefore, it is still possible that human basophils may acquire an APC function under specific conditions, and as for neutrophils and eosinophils this would likely be linked to inflammatory situations or pathogenesis.

## Concluding Remarks

Evidently, granulocytes are more than just the first line of defense internalizing and killing pathogens. These cells can present antigens under certain conditions and may orchestrate adaptive immunity. While an APC function has been identified in these cells, the underlying molecular mechanisms resulting in antigen presentation and the clinical significance still remain unclear. Understanding the extent of granulocyte contribution to the magnitude and quality of adaptive immunity *via* antigen presentation would help decipher their exact role in prevention or contribution to pathological responses in diseases. Defining their role may also provide strategies for development of novel vaccines and/or therapeutics *via* manipulating or targeting granulocytes.

## Author Contributions

All authors listed have made a substantial, direct, and intellectual contribution to the work and approved it for publication.

## Conflict of Interest Statement

The authors declare that the research was conducted in the absence of any commercial or financial relationships that could be construed as a potential conflict of interest.
